# Crystal Structure of *Borrelia turicatae* protein, BTA121, a differentially regulated  gene in the tick-mammalian transmission cycle of relapsing fever spirochetes

**DOI:** 10.1038/s41598-017-14959-9

**Published:** 2017-11-10

**Authors:** Zhipu Luo, Alan J. Kelleher, Rabih Darwiche, Elissa M. Hudspeth, Oluwatosin K. Shittu, Aparna Krishnavajhala, Roger Schneiter, Job E. Lopez, Oluwatoyin A. Asojo

**Affiliations:** 1 Synchrotron Radiation Research Section, Macromolecular Crystallography Laboratory, National Cancer Institute, Argonne, Illinois 60439 USA; 2 National School of Tropical Medicine, Baylor College of Medicine, Houston Texas, United States of America; 3 Division of Biochemistry, Department of Biology, University of Fribourg Chemin du Musée 10, CH 1700, Fribourg, Switzerland

## Abstract

Tick-borne relapsing fever (RF) borreliosis is a neglected disease that is often misdiagnosed. RF species circulating in the United States include *Borrelia turicatae*, which is transmitted by argasid ticks. Environmental adaptation by RF *Borrelia* is poorly understood, however our previous studies indicated differential regulation of *B. turicatae* genes localized on the 150 kb linear megaplasmid during the tick-mammalian transmission cycle, including *bta121*. This gene is up-regulated by *B. turicatae* in the tick versus the mammal, and the encoded protein (BTA121) is predicted to be surface localized. The structure of BTA121 was solved by single-wavelength anomalous dispersion (SAD) using selenomethionine-derivative protein. The topology of BTA121 is unique with four helical domains organized into two helical bundles. Due to the sequence similarity of several genes on the megaplasmid, BTA121 can serve as a model for their tertiary  structures. BTA121 has large interconnected tunnels and cavities that can accommodate ligands, notably long parallel helices, which have a large hydrophobic central pocket. Preliminary *in-vitro* studies suggest that BTA121 binds lipids, notably palmitate with a similar order of binding affinity as tablysin-15, a known palmitate-binding protein. The reported data will guide mechanistic studies to determine the role of BTA121 in the tick-mammalian transmission cycle of *B. turicatae*.

## Introduction

Relapsing fever (RF) spirochetes cause recurrent febrile episodes, uncontrollable chills, nausea, vomiting, miscarriage, and potential death if untreated^1,2^. In parts of Africa, RF spirochetes are in the top five causes of hospital admissions and child morbidity and mortality, while in Central Asia, North America, and Latin America the disease is overlooked^3–13^. The nonspecific symptoms often results in the disease’s misdiagnosis as malaria, and consequently the pathogens are underreported^14^. Additionally, *Borrelia myiamotoi* is a recently recognized emerging pathogen^15–21^, and the refugee crisis in the Middle East has caused the re-emergence of louse-borne RF spirochetes in Europe^22,23^. With the global impact of RF spirochetes there is a need for the development of strategies to interrupt the pathogen’s life cycle.

While both argasid and ixodid ticks transmit RF spirochetes, the pathogen’s life cycle is more defined in the argasids. The spirochetes initially enter and colonize the tick midgut, and within two weeks after acquisition a population will migrate and colonize the salivary glands. RF spirochetes are transmitted to the vertebrate host within seconds of tick bite, indicating that spirochetes colonizing the salivary glands are essential for establishing infection^24^. Within the mammal, infection is characterized by two events, early and persistent infection. Persistent infection is driven by antigenic variation, while the molecular events characterizing early infection remain vague.

Recent work has indicated that the linear megaplasmids of RF spirochetes likely have important roles in vector colonization and establishing early mammalian infection^25^. The *Borrelia turicatae-Ornithodoros turicata* model showed that a majority of genes located on the 150 kb linear megaplasmid were up-regulated in tick-like *in vitro* growth conditions (growing the bacteria at 22 °C). Further evaluation of a cluster of genes toward the 3′ end of the plasmid confirmed their up-regulation in the tick compared to mammalian blood^25^. Evaluation of primary amino acid sequences indicated that homologues were absent outside the *Borrelia* genus, thus most of these proteins are classified as domains of unknown function (DUFs). This limitation has significantly hindered progress in the understanding of the molecular mechanisms of RF spirochete pathogenesis.

One of the proteins identified in these studies was classified as BTA121. While BTA121 does not share appreciable sequence similarity to any known protein domains or motifs, its N-terminal region is enriched in proline, glycine, and glutamic acid and contains a potential procyclic acidic repetitive protein (PARP) domain that was previously reported as having roles in *Trypanosoma brucei* survival and successful replication in its insect vector, the tsetse fly^26^. It is possible that the putative PARP in BTA121 plays similar roles but its structure remains unknown as it is disordered in all structures of proteins containing it. Efforts to clarify the function of BTA121 include determining if it shares any structural similarity with proteins of known function as these may offer insights towards possible functions. Towards these ends we expressed, purified, and solved the crystal structure of BTA121 and present here the results of these studies.

## Results

### Recombinant BTA121

N-terminal dodeca-histidine (dodeca-His) tagged BTA121 (BTA121-His) was produced as a soluble protein in *Escherichia coli*. Selenomethionine derivatized BTA121-His was also produced for phasing. Recombinant BTA121 included the putative PARP but lacked the signal peptide. The dodeca-His tag was cleaved using TEV-protease to yield ~95% pure BTA121 as assessed by electrophoresis and size exclusion chromatography (Figures S1 through S4). Successful cleavage was confirmed by Western blotting (Figure S1). Recombinant BTA121 migrates predominantly as a monomer on both reducing and non-reducing Coomassie stained SDS-PAGE gel (Figure S2).

### Structure determination

BTA121-His did not crystallize whereas the untagged protein gave large crystals within 46 hours in high salt conditions. Both native and selenomethionine derivative BTA121 crystals diffracted to less than 3.5 Å at home source. Two higher resolution data sets were collected at the Advanced Photon Source (Argonne National Laboratory, Argonne, USA) at 100 K using selenomethionine derivative BTA121 crystals. Single-wavelength anomalous dispersion for Se (Se-SAD) phases were calculated and used to generate initial experimental maps from a 3.2 Å resolution data collected at 0.979 Å wavelength (Table 1). The structure model was built using automatic building and refinement into these maps. The final refined structure was obtained by phase extension by molecular replacement into the 2.8 Å resolution data collected at 1.000 Å wavelength. Refined model coordinates and structure factors have been deposited in Protein Data Bank (PDB) as entry 5VJ4. Data collection and structure refinement statistics are listed in Table 1.

**Table 1:** Statistics for data collection and model refinement.

	**BTA121**	
**Data Collection**	**PDB entry 5VJ4**	
X-ray Source	SER-CAT APS-22BM	SBC-CAT APS-19ID
Detector	MAR225	Pilatus 6 M
Wavelength (Å)	1.000	0.979
Space group	*P*6_1_	*P*6_1_
Cell dimensions	a = b = 102.06 Å, c = 183.21 Å α=β = 90.00°, γ = 120°	a = b = 102.60 Å, c = 184.70 Å α = γ = 90.00°, β = 120 °
Resolution (Å)	50.00 –2.80 (2.90 –2.80)	50.00 –3.2 (3.31 – 3.20)
Number of total reflections	152,863	368,989
Number of unique reflections	26,628 (2,665)	18,312 (1,822)
*R*_*merge*_ (%)	8.1 (116.1)	12.8 (202.2)
*I/σ(I)*	20.3 (1.7)	31.0 (1.7)
Completeness (%)	100.0 (100.0)	100.0 (100.0)
Redundancy	5.7 (5.7)	20.2 (20.5)
CC_1/2_	0.941 (0.922)	0.940 (0.899)
Wilson B-factor (Å^2^)	76.2	99.7
**Refinement** (REFMAC-5)		
Resolution (Å)	39.80–2.80 (2.90–2.80)	
*R*_*work*_	0.196 (0.365)	
*R*_*free*_	0.224 (0.340)	
r.m.s. deviation bond length (Å)	0.011	
r.m.s. deviation bond angles (°)	1.466	
MolProbity analysis		
Ramachandran outliers	0.55%	
Ramachandran favored	96.88%	
No. of non-H protein atoms	4396	
No. of water molecules	7	
Ions (SO4^2−^)	12	
Correlation coefficient *F*_*o*_ *-F*_*c*_	0.958 (0.949)	
Average B-factors (Å^2^)	83.0	

### Overall Structure of BTA121

**Figure 1:**
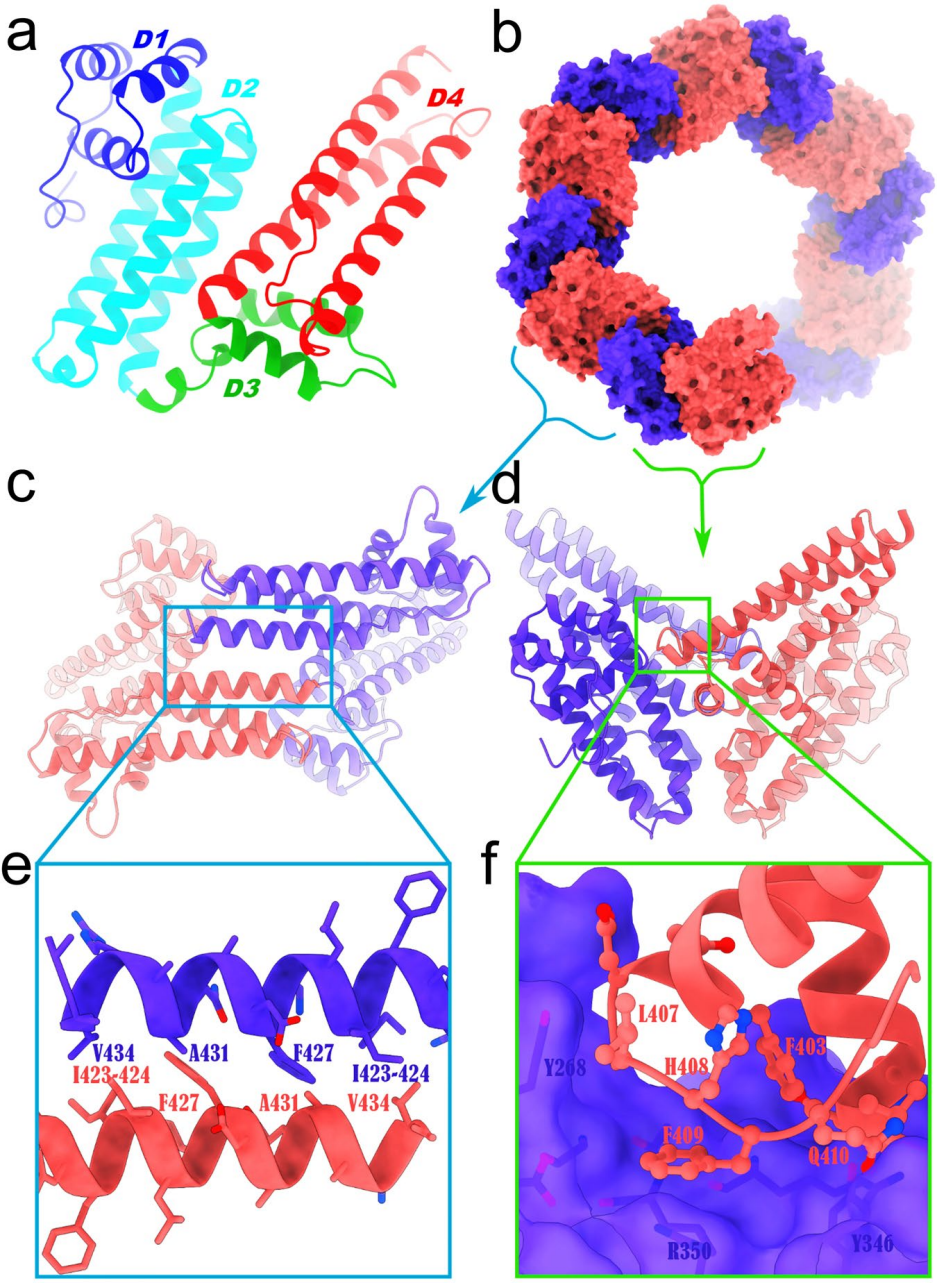
Structure and packing of BTA121. (**a**) BTA121 consists of four domains (D1, blue, 164–214; D2, cyan, 215–299; D3, green, 300–344; D4, red 345–435) and each domain has three alpha helices. (**b**) There are two BTA121 monomers in the asymmetric unit colored as blue and salmon forming two possible crystallographic dimers (**c**) dimer 1 and (**d**) dimer 2. (**e**) Close up of intermolecular interactions of dimer 1 reveals a palindromic sequence (side chains shown as sticks). (**f**) Details of intermolecular interactions of dimer 2.

A total of 548 amino acid residues and 13 sulfate ions have ordered electron density  forming  a dimer. The first 91 amino acids of BTA121 and N-terminal putative PARP domain are disordered for each monomer. The disorder of the PARP may contribute to the lower resolution of the over-all structure. More studies are required to determine if the disordered residues will be stabilized by co-crystallization with small molecules identified in the tick saliva. BTA121 is a completely helical protein separated into an N-terminal and a C-terminal helical bundle. The monomer can also be viewed as having four helical domains (D1, 164–214; D2, 215–299; D3, 300–344; and D4, 345–435). Domains D1 and D2 are in the N-terminal bundle while D3 and D4 are in the C-terminal bundle (Fig. 1a). The refined model has two monomers of BTA121 in the asymmetric unit and crystal packing in the unit cell reveals a pore like formation with two possible crystallographic dimers of BTA121 (Fig. 1b).

There are two possible crystallographic dimers; dimer 1 has an interface of parallel helices (Fig. 1c and e); while dimer 2 interfaces through loops (Fig. 1d and f). The interactions at the dimer 1 interface are hydrophobic and have a palindromic sequence (Fig. 1e). It is unclear if either dimer is biological and analysis using the protein interfaces, surfaces and assemblies service PISA^27^ at the European Bioinformatics Institute (http://www.ebi.ac.uk/pdbe/prot_int/pistart.html) suggests that dimerization is an artifact of crystallization and neither dimer has appreciable complex formation significance score (CSS over 0.03) whereas a real dimer has a CSS closer to 1. Furthermore size exclusion chromatography reveals that there are both monomer and dimer species in solution.

**Figure 2:**
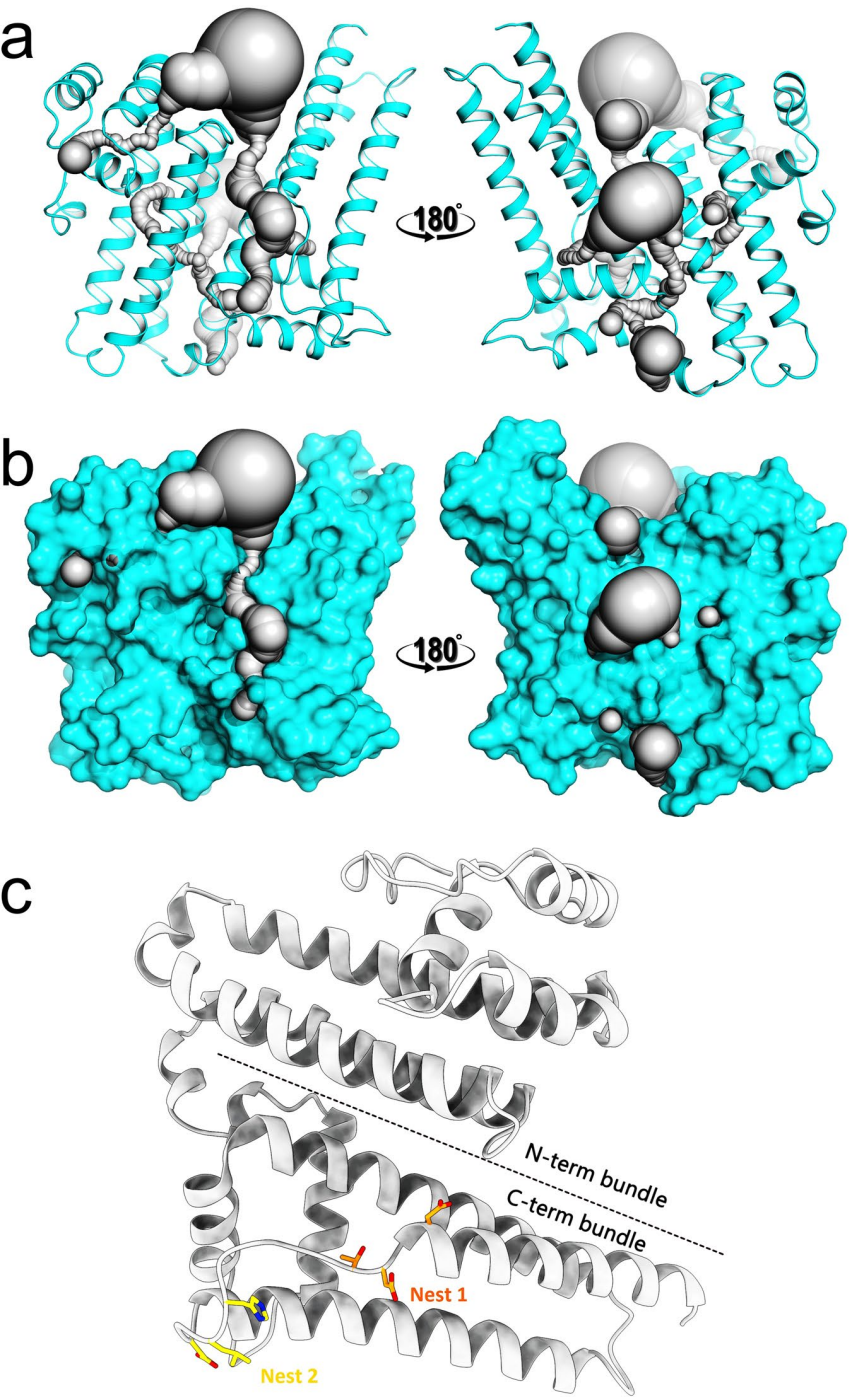
BTA121 monomer has tunnels, and nests. (**a**) Ribbon diagram of BTA121 in cyan with the interconnected tunnels and cavities shown as gray surface. (**b**) Surface representation of the same view of BTA121 in cyan with tunnels and cavity in gray. (**c**) BTA121 monomer in gray location of the top two nests and the helical bundles are shown.

The BTA121 monomers are very similar with rmsd of 0.388 Å for alignment of main chain atoms. The most variable regions between the monomers are loop regions. CAVER^28,29^ analysis reveals that the protein has several interconnected tunnels and cavities (Fig. 2a and b). The volumes of the largest ten cavities were calculated using ProFunc^30,31^ and were revealed to be as large as 5212 Å^3^ (Table 2). The channels and tunnels connect all the cavities containing sulfate ions. The presence of such an extensive network of tunnels and cavities suggests that BTA121 may bind small molecular weight ligands, including lipids.

**Table 2:** Cavities in BTA121.

Cleft #	**Volume** (Å^3^)	**Ligands**
1	5212.27	4 sulfate ions
2	3427.31	3 sulfate ions
3	3520.12	1 sulfate ion
4	1452.52	1 sulfate ion
5	1169.86	2 sulfate ions
6	725.20	1 sulfate ion
7	1027.69	
8	891.84	1 sulfate ion
9	612.98	
10	554.34	

### Lipid binding by BTA121

**Figure 3:**
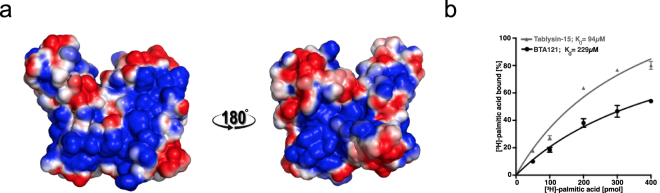
Lipid-binding by BTA121 (**a**) Hydrophobic patch (in-blue) on BTA121 surface shown in the same orientation as Fig. 2a and b. (**b**) The *in vitro* palmitate-binding  affinity of BTA121 is of similar order of magnitude as the palmitate-binding protein tablysin-15.

Among the large cavities on BTA121 are cavities between long parallel helices, which have hydrophobic pockets (Fig. 3A). Due to the presence of hydrophobic pockets and the prior observation that lipid-binding proteins have varied structural topology^32^, the possibility that BTA121 would bind lipids was investigated. The positive control used is a known lipid-binding protein tablysin-15, a protein present in the saliva of the horsefly *Tabanus yao*, which scavanges cysteinyl leukotriene, an eicosanoid lipid^33,34^. Tablysin-15 belongs to the SCP/TAPS (Sperm-coating protein/Tpx/antigen 5/pathogenesis related-1/Sc7) superfamily and palmitate binding has been confirmed using our *in vitro* assay for other members of the superfamily^35–37^. Using this same *in vitro* assay, BTA121 binds palmitate with micromolar K_d_, which is in the order of magnitude of palmitate binding by tablysin-15 (Fig. 3B).

## Discussion

### Comparison of BTA121 to other protein structures

In order to determine if BTA121 shares structural similarity to any reported structures, PDBFold (http://www.ebi.ac.uk/msd-srv/ssm)^38^ analysis was performed using the default threshold cutoffs of 70% for the percentage of secondary structure of target chain identified in the query protein and of the secondary structure of query chain. No matches were identified against 130,141 entries in the PDB at the default cut-off and 9 highly helical proteins matches were identified as possible matches only upon lowering the threshold to 40%. None of the matches share over 9% sequence identity to BTA121 and the length of aligned residues varied between 79 and 130 amino acids of the 275 present in the monomer. The most similar structure is the Lyme disease causing spirochete outer surface protein BBA65 from *Borrelia burgdorferi*. None of the matches from PDBFold analysis have known function, so no functions for BTA121 could be proposed from the analysis. BTA121 does not share any appreciable sequence or tertiary structural identity to the only reported *B. turicatae* structures in the PDB of neurotropism associated variable surface protein (VSP)^39^.

### Functional Prediction For BTA121

The structure of BTA121 was submitted to the ProFunc^30,31^ analysis to identify possible structural and functional motifs. Nest analysis was performed to identify structural motifs found in functionally important regions of BTA121, and two were found that were significant (Table 3). Both Nests are located in the C-terminal helical bundle (Fig. 2c). The BTA121 structure shares very low sequence identity to any protein structure in the PDB. The closest structure is that of carnation mottle virus (PDB entry 1OPO) with 15% sequence identity and the structures are completely different as the virus structure is made up entirely of beta strands. There were no certain, probable, or possible hits identified for any of the 3D functional analyses and BTA121 does not contain any of the 584 known enzyme active site templates. The only DNA-binding template identified from 5320 DNA-binding templates (PDB entry 1FOS), did not align with BTA121 structurally due to too many gaps in the sequence alignment. Likewise a reverse template comparison with all structures in the PDB identified hits with meaningless alignment and many gaps and insertions. These bioinformatics tools did not predict any possible function, however, the large hydrophobic cavities present in BTA121 suggested lipid-binding ability. Thus *in vitro* palmitate binding assays were conducted which revealed that BTA121 had palmitate-binding affinity similar to that of a known lipid binding protein tablysin-15. More studies are needed to determine how BTA121 binds palmitate as well as to identify if it binds other lipids with tighter affinity. The discovery of the palmitate-binding ability of BTA121 offers a functional activity that can be tested to determine the underlying mechanisms for BTA121 in the pathogen life cycle. Given that tick saliva contains diverse small molecules including lipids derivatives^40–42^ that could bind to a palmitate-binding site in BTA121, we can begin to test possible mechanisms by which the pathogen exploits the tick-salivary compounds for transmission.

**Table 3:** Nests in BTA121.

**Nest**	**Score**	**Residue range**	**Cleft #**
1.	3.60	D335 -H338	1
2.	2.36	T343 -D346	2

### The *B. turicatae* megaplasmid proteins

The structure of BTA121 is the first of the members of the megaplasmids of RF. A blast analysis revealed that these proteins share high sequence similarity (up to 90%). ESPript^43^ alignment of these proteins revealed that they all share extensive similarity in the C-terminal helical bundle and more differences in their N-termini (Figure S5). Several helices are well conserved without any gaps or insertions, notably α1, α2, α5, η1, η2, α8, α9, η4, α10. Overall the locations of the helices are conserved for the proteins, which indicates that BTA121 can serve as a homology model for several RF spirochete proteins. Interestingly, the nest regions are not well conserved for all the proteins, which may suggest that they all have different substrates or ligands.

## Conclusion

Recombinant BTA121 was produced and the crystal structure of BTA121 was solved using Se-SAD phasing. This is the first structure of any of the proteins in megaplasmid likely responsible for spirochete survival in the tick vector. BTA121 does not share structural similarity with known proteins, and has the characteristic palmitate-binding cavity observed in tablysin-15, and palmitate binding was confirmed *in vitro*. BTA121 has a network of interconnected tunnels connecting the large cavities some of which contain sulfate ions from the crystallization solution. Future studies are required to determine how the structure of BTA121 mediates its possible roles in vector adaptation.

## Methods

### Cloning, and expression BTA121

The amino-acid sequence of full-length *bta121* excluding the signal peptide but including the putative PARP domain was used as a template for the chemical synthesis of DNA expression constructs to encode the protein (without the signal peptide) with a N-terminal dodeca-His tag, followed by a TEV protease cleavage (GenScript USA Inc., Piscataway, New Jersey, USA). Additional residues that were not conducive to protein solubility in the amino-terminus were also removed and the amino-acid sequence for the expressed protein is shown in Figure S5. The synthetic construct was codon-optimized for expression in *Escherichia coli* and cloned into the NdeI / XhoI, sites of pET-19b vector (Novagen, Madison, Wisconsin, USA). The plasmid was transformed into BL21-CodonPlus-(DE3)-RIPL (Stratagene) by heat shock. Positive colonies were identified by colony PCR with T7 promoter and T7 terminator primers (EMD Millipore, Rockland, Massachusetts, USA). A single positive colony was selected, verified, and used for large-scale expression. A 1.6 L culture was grown from a 25 mL overnight starter culture using 50 μg/L of kanamycin. Protein induction was initiated by the addition of IPTG to a final concentration of 0.5 mM, for 16 h at 28 °C, using NZYM media. Selenomethionine labeled protein was expressed using selenomethionine medium Complete (molecular dimensions, USA) instead of NZYM and growing for 20 h at 28 °C post induction. Cell pellets were harvested by centrifugation at 8000 g for 10 min and stored at -80 °C until used.

### Protein Purification

Cell pellet from 800 mL culture was resuspended in lysis buffer (100 mL of phosphate buffered saline (PBS) pH 7.4, 10% (v/v) Glycerol, 5% (v/v) Triton-X 100, 0.05% (v/v) beta-mercaptoethanol, and one Roche complete EDTA-free protease inhibitor tablet), and lysed under high pressure in an Emulsiflex homogenizer (Avestin Canada). The supernatant was clarified by centrifugation at 8000 g for 10 min prior to purification by immobilized affinity chromatography (IMAC) using two 5 mL Histrap-FF crude column (GE Healthcare, Piscataway, NJ), an AKTA purifier (GE Healthcare, Piscataway, NJ) and PBS pH 7.4 with 20 mM imidazole as the binding buffer. Nonspecifically bound proteins were removed by extensive washing with binding buffer prior to elution with an imidazole gradient.

Fractions containing purified BTA121 were identified visually by electrophoresis on reduced Novex NuPAGE MES gels. These fractions were pooled and the concentration of protein was assessed by absorbance at 280 nm. The N-terminal dodeca His tag was cleaved using recombinant hexa histidine tagged TEV protease. For each 0.1 mg of BTA121, 5 μg of recombinant TEV protease^44^ was added and incubated for 12 hours at 4 °C. This was followed by dialysis into PBS pH 7.4 to remove imidazole and the TEV protease storage buffer. A final reverse capture purification step from a 5 mL Histrap-FF crude column using PBS pH 7.4 was used to generate untagged BTA121. Selenomethionine labeled protein was purified in an identical fashion as unlabeled protein. The resulting protein was dialyzed into 50 mM Tris pH 7.8 and stored at -80 °C until used.

### Western Blot

1 μg of BTA121 protein with and without dodeca-His tag was electrophoretically separated and transferred to PVDF membrane using TGX gels, Mini-PROTEAN Tetra cell, and the Mini Trans Blot system (BioRad, Hercules, California, US). The PVDF membrane was subsequently probed with a monoclonal anti-poly His-peroxidase antibody (Sigma-Aldrich, St. Louis, Missouri, US) at a 1:4000 dilution, to determine successful removal of the dodeca-His tag.

### Size exclusion chromatography

A Phenomenex Yarra 3 µ SEC-2000 HPLC size exclusion column (SEC) was used with an in-line Phenomenex SecurityGuard™ Cartridge for all samples, except that of BTA121 the guard cartridge was not present. The column was equilibrated with the appropriate mobile phase on the HPLC system, which was a Shimadzu Prominence Ultra-Fast Liquid Chromatography (UFLC) connected to a Shimadzu SPD-M20A photo diode array detector (PDA). Data was collected and analyzed with LC Solution software (version 1.25, Shimadzu). Protein samples were prepared by diluting to a concentration of 1.0 mg/mL with the appropriate mobile phase. A total of 50 µg was filtered through a 0.22 µm membrane and loaded directly onto the column using the auto-sampler. The method was run at room temperature at a flow rate of 0.5 mL/min for 40 minutes. Absorbance from 190 nm to 800 nm at a frequency of 1.5625 hertz was measured using the PDA. The 280 nm wavelength chromatographs (A280) were extracted to evaluate the presence of proteins. A 3-dimensional plot of wavelength and absorbance versus retention time was plotted to confirm that A280 peaks had a protein spectra and to evaluate the presence of non-protein species. The molecular weight of the protein peaks were estimated using a Bio-rad gel filtration standard protein mixture as described in supplemental methods.

### *In vitro* lipid-binding assay

To determine palmitate binding *in vitro*, a radioligand-binding assay was performed as described previously^45,46^. Purified proteins (100 pmol) in binding buffer (20 mM Tris, pH 7.5, 30 mM NaCl, 0.05% Triton X-100) was incubated with [^3^H]-palmitic acid (0–400 pmol) for 1 h at 30 °C. The protein was then separated from the unbound ligand by adsorption to Q-sepharose beads (GE Healthcare); beads were washed with washing buffer (20 mM Tris pH 7.5), proteins were eluted (20 mM Tris, pH 7.5, 1 M NaCl), and the radioligand was quantified by scintillation counting. To determine non-specific binding, the binding reaction was performed without the addition of protein into the binding assay.

### Crystallization, Data Collection and Structure Determination

Both BTA121 and selenomethionine labeled BTA121 crystals were grown by vapor diffusion by equilibrating a 2 μL drop containing equal volumes of 28 mg/mL protein and precipitant (1.8 M lithium sulfate, 50 mM sodium HEPES pH 7.5) against a well containing 300 μL of precipitant. The crystals were briefly immersed in cryo-protectant (1.8 M lithium sulfate, 50 mM sodium HEPES pH 7.5 with 20% (v/v) 2-methyl-2,4-pentanediol) and flash cooled directly in a liquid N_2_ stream prior to data collection. X-ray diffraction data were collected at 100 K at the Advanced Photon Source (Argonne National Laboratory, Argonne, USA). The Se-SAD data were collected at wavelength of 0.979 Å at the SBC-CAT beamline 19-ID with Pilatus 6 M detector. The best diffraction data were collected at wavelength of 1.000 Å at SER-CAT beamline 22-BM with a MAR225 CCD detector. The diffraction data were processed with HKL2000^47^.

Initial experimental maps were calculated within Phenix using the Se-SAD phases generated with Phenix.autosol. After automatic model building with Phenix.autobuild an initial 406 residues homo-dimer model with *R* = 0.31 and *R*_*free*_ = 0.35 was generated^45,46^. The structure was improved by using the more complete chain as a molecular replacement search model against the best diffraction data at 2.8 Å in PHASER^48^. The final model was obtained by iterative manual building in Coot and refined using REFMAC5^49,50^ and PHENIX^51^. Data collection and structure refinement statistics are listed in Table 1. Figures 1 and 2c were generated using ChimerX^52^ while other figures were generated using PyMOL^53^.

## Electronic supplementary material

Supplementary information
