# Supplementary material for: Crystal Structure of Borrelia turicatae protein, BTA121, a differentially regulated gene in the tick-mammalian transmission cycle of relapsing fever spirochetes
Source: Sci Rep. 2017 Nov 10;7:15310. doi: 10.1038/s41598-017-14959-9 (PMC5681642; doi:10.1038/s41598-017-14959-9)
Supplement: Supplementary file 1 — Supplementary information [file 41598_2017_14959_MOESM1_ESM.pdf]

Supplementary Information for Crystal Structure of *Borrelia turicatae* protein, BTA121, a differentially regulated gene in the tick-mammalian transmission cycle of relapsing fever spirochetes.

Zhipu Luo[1], Alan J. Kelleher[2], Rabih Darwiche[3], Elissa H. Hudspeth[2], Oluwatosin K. Shittu[2], Aparna Krishnavajhala[2], Roger Schneiter[3], Job E. Lopez[2] and Oluwatoyin A. Asojo[2*]

[1] Synchrotron Radiation Research Section, Macromolecular Crystallography Laboratory, National Cancer Institute, Argonne, Illinois 60439, USA

[2] National School of Tropical Medicine, Baylor College of Medicine, Houston Texas, United States of America

[3] Division of Biochemistry, Department of Biology, University of Fribourg, Chemin du Musée 10, CH 1700 Fribourg, Switzerland

***Running Title*: Crystal Structure of BTA121**

Keywords: relapsing fever; neglected tropical diseases; vector adaptation; lipid binding; tick borne diseases; structure-function relationships; bioinformatics; argasids ticks

Correspondence to: asojo@bcm.edu (OAA)

Purification of BTA121

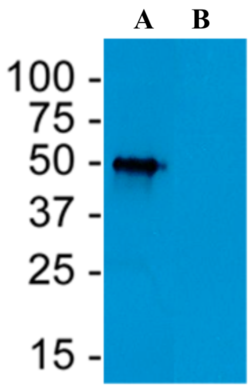

Figure S.1: Confirmation of cleavage of Histag by anti-His Western blotting. Lane A contains BTA121-His while Lane B contains BTA121 post removal of the Histag.

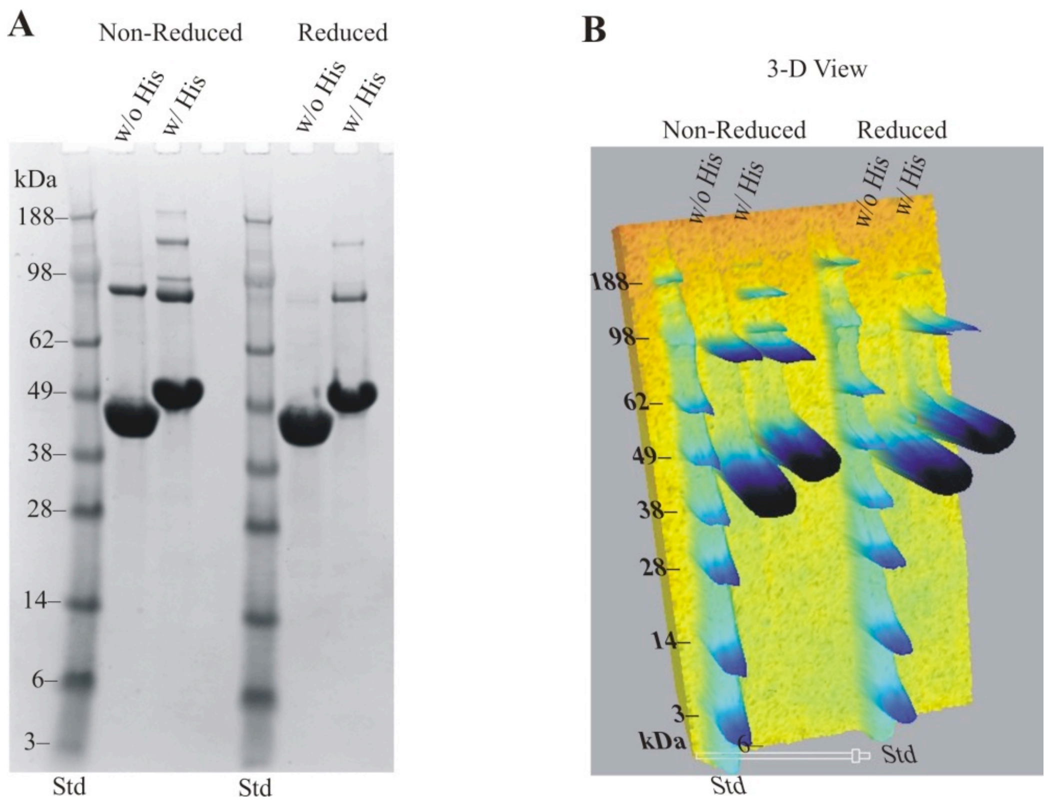

Figure S2: A) SDS-page gel showing purity of BTA121, visualized by Coomassie blue staining. Electrophoretic mobility on the reduced gel is consistent with the theoretical molecular mass for the tagged and untagged proteins. B) 3D gel of Fig S.2A visualized by false color.

Size exclusion chromatography

The molecular mass of the eluted peak is determined using a calibration curve based on known standards, not just by visualization. The standard consisted of bovine thyroglobulin (670 kDa), bovine γ-globulin (158 kDa), chicken ovalbumin (44 kDa), horse myoglobin (17 kDa), and vitamin B_{12} (1.35 kDa), which was injected onto the column using the same method as the BTA121 samples. For samples analyzed on different days or with different column/guard cartridge set-ups, a gel filtration standard was injected on the same day and with the same set-up as the experimental sample. In addition, blank injections of the mobile phases were used to confirm a stable baseline and the absence of carry-over (prior injected sample eluting during the subsequent injection run). Blue dextran was injected to estimate the retention time of the void volume (the volume present from the injection loop to the PDA detector, excluding the pore volume of the beads). This provides the retention time at which a compound will elute that is larger than the pore size of the beads (145 μm diameter) and has no interaction with the bead surface. A 1 M sodium chloride (NaCl) solution was injected to estimate the pore volume of the beads. The retention times of the protein standards and blue dextran were utilized to calculate the distribution coefficient (K_d) for each standard and to create a plot of the log of the molecular weight (MW) versus K_d. Normalizing the retention times to K_d removes system and column specific information (void and column volume) allowing direct comparison of the data to that obtained from other systems and columns. The slope and y-intercept of a best-fit linear line was used to calculate the molecular weight of the sample peaks.

Kd (distribution coefficient) = (Ve - Vo) / (Vt - Vo)

Ve = elution volume of sample

Vo = void volume (blue dextran)

Vt = total column volume (3.14*radius^2

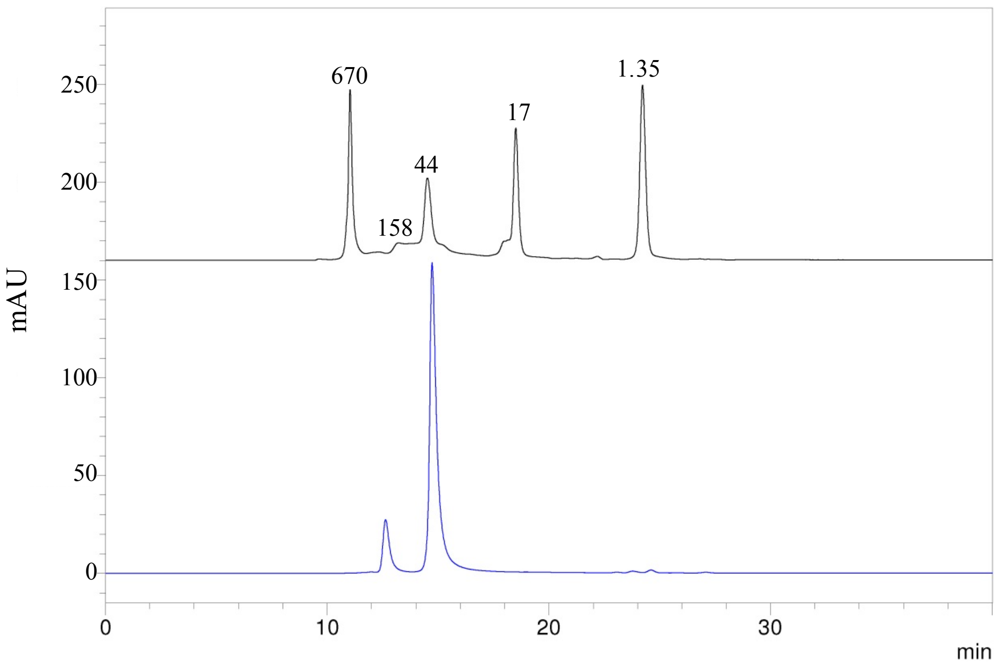

Figure S.3 Purity of freshly thawed untagged BTA121 reveals two peaks. These can be separated by gel filtration, however removing the minor peak does not improve the quality of the crystals. Similar profile was observed for the selenomethionine protein.

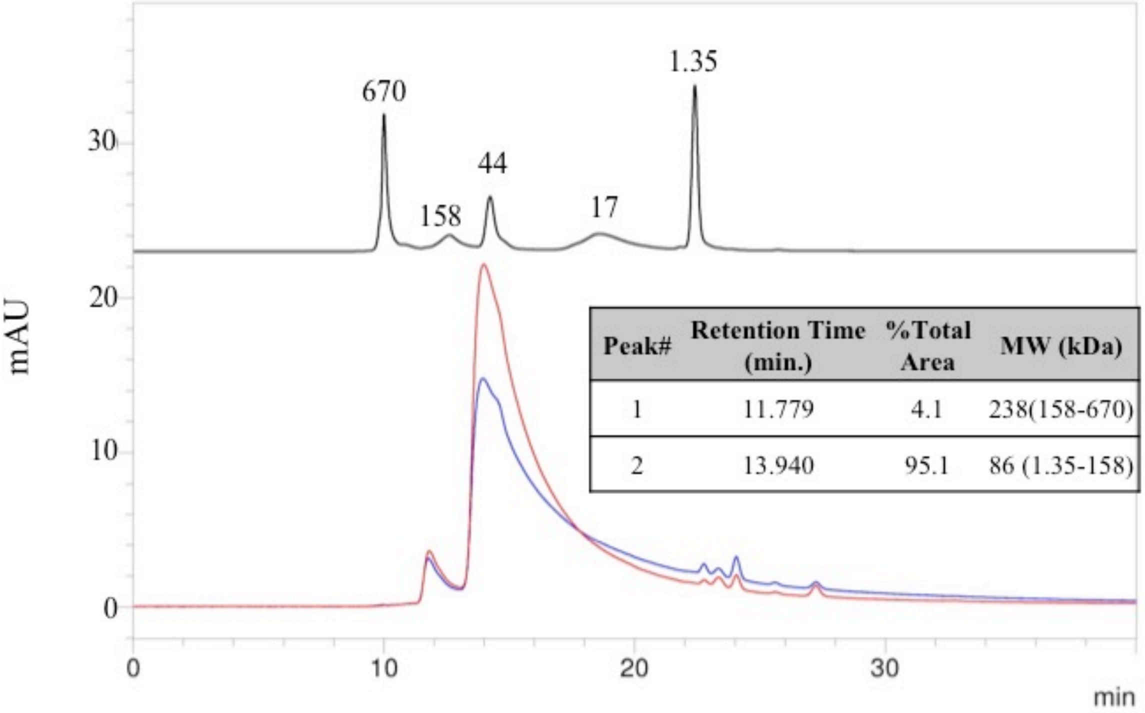

Peak#	Retention Time (min.)	%Total Area	MW (kDa)
1	11.779	4.1	238(158-670)
2	13.940	95.1	86 (1.35-158)

Figure S.4: Extensive peak broadening occurs after storing BTA121 at 4°C. The sample stored at 28 mg/mL for greater than 70 days (red) has a consistent profile with the sample stored at 5 mg/mL for 43 days (blue). Since 100 μg was injected of the sample stored at 28 mg/mL and 50 μg was injected of the sample stored at 5 mg/mL, the chromatograph of the 28 mg/mL sample was adjusted 2-fold for comparison.

Figure S.5. Structural and primary sequence alignment of BTA121 with other *B. turicatae* proteins from the megaplasmid. This figure was generated with ESPript[1]. The different secondary structure elements shown are alpha helices as large squiggles labelled (a), 3_{10}-helices as small squiggles labelled (h), beta strands as arrows (b), and beta turns (TT). Identical residues are shown in white on red background, and conserved residues in red. The locations of the cysteine residues involved in disulfide bonds are numbered in green.

1 Gouet, P., Robert, X. & Courcelle, E. ESPript/ENDscript: Extracting and rendering sequence and 3D information from atomic structures of proteins. *Nucleic Acids Res* **31**, 3320-3323 (2003).

```
fBTA121
             1        10        20        30        40        50        60
fBTA121      MLKIKCFSLLLVLTLLLLLVISCDSGSKAVAGTDTETEAVTNTGAGLEAGVRPGVGLRIS
BTA121       ............................................................
BTA121-His   ....................................................HHHHHHHH
BTA116       MLKIKCFSLLLILILLLLLFISCDLGIKARTNTRA.........................
BTA123       MLKIKCFSLLLVLILLLLLFISCDSGSKAGAGTNTGAGGGAGSG..TNT...........
BTA126       MVKIKCFSVLLVLILLLLLVISCDSGSKAGAGTNTRAVVKSVAG..TNTRAVVKS.....
BTA127       MLNIRHISVLLVLIFLLFLFINCDSRSKAGAGTNTGAGGGAG..................
BTA119       MVKIKCFSLLLVLILLLLLVISCDSGSKAGAGTNTGITTGKTTK..ITTGTNT.......
BTA118       MVKIKCFSLLLVLILLLLLFISCDSGSKAGAGTNTGITTGKTTK..ITTGTTTGT.....
BTA117       MLKIKCFSLLLVLILLLLLVISCDSGSKAGAGTNTVTDTGKTTK..ITTGTTTGT.....
BTA122       MLKIKCFSLLLVLILLLLLFISCDSGSKAGAGT...........................
BTA124       MVKIKCFSLLLVLIFLLLLVISCGSGSKAVTNT......GKTTG..TNTGITTGT.....
BTA120       MLKIKCFSLLLVLILLLLLVISCDSGSKAGAGTNTVTDTGKTTG..TNTG..........
BTA115       MVKIKCFSLLLILILLLLLFISCDSGIKAGTNTKTEVKPGAG....LDAVVKPGAGLDAV
BTA112       MLKVRYRNSLLVLILLLLLVMGCNFKSPKDAAASGENSIVKKNE................
BTA129       MMKLSHFSLFLVLIFILLLVVNCDLKSKGSTGIEENL.TSKREVKK..............
```

```
fBTA121
                      70        80        90       100       110       120
fBTA121      TVPVVVIRLESGVRPESGVGPESGVGPEAGVGPEAGVGPESGVGPEAGVGPEAGVGPESG
BTA121       ..........GGVRPESGVGPESGVGPEAGVGPEAGVGPESGVGPEAGVGPEAGVGPESG
BTA121-His   HHHHENLYFQGGVRPESGVGPESGVGPEAGVGPEAGVGPESGVGPEAGVGPEAGVGPESG
BTA116       ............................................................
BTA123       ............................................................
BTA126       .................GSGTNTR.................................TVVE
BTA127       ............................................................
BTA119       ............................................................
BTA118       .................TTGTTT.....................................
BTA117       .................NTGTNTGTNTGTNTGTTTGTN..........TGTNTG..TNTG
BTA122       ............................................................
BTA124       .....................................................NTG..ITTG
BTA120       ............................................................
BTA115       VV.....KP.......GAGLEAGAGLGAGE...AGLG.AGGAGLEAGEAGEAG......
BTA112       ............................................................
BTA129       ...........................................LG.................
```

```
                                                                     α1
fBTA121
                     130       140       150       160       170       180
fBTA121      VGPESGVGPEAGVRPEAGVRPEAGAGTDTETEEEIEEVVGDEEALAYLNETVIDPKLIAL
BTA121       VGPESGVGPEAGVRPEAGVRPEAGAGTDTETEEEIEEVVGDEEALAYLNETVIDPKLIAL
BTA121-His   VGPESGVGPEAGVRPEAGVRPEAGAGTDTETEEEIEEVVGDEEALAYLNETVIDPKLIAL
BTA116       .............................ELRTKAEEIVGDEEELAYLDDI.SDPKLKAL
BTA123       ...............................GVGAGGGAELKDEDELAYLEDKVIDPELMAL
BTA126       SGSDKELG......AVTGIDERTEPKPEEETGLITDVEVKDEDELAYLNETVIDPRFQAL
BTA127       ........................SGINTRPEPNTEVEVGDEEALAYLEEKVTDSKLKAL
BTA119       ........................GTNTGTNTVTDVEVGDEEALAYLNETVTYPKLKEL
BTA118       ........G.....TNTGITTETNTGTTTGTNTVTDVEVGDEEALAYLNETVTYPKLKEL
BTA117       TNTGTNTG......TNTGTNTGTNTGTNTGTNTVTDVEVGDEEALAYLEDKVTYPKLIAL
BTA122       ...............................DTETEVGDEEALAYLEDKVTYPKLKEL
BTA124       TNTEITTG......TNTEITTGTNTGTNTGTNTGTDVEVGDEEGLAYLEGKVTDPRVQAL
BTA120       ........................TNTG..TDTEAEVGDEEALAYLEDI.SDSKLKEL
BTA115       .....EAGLEA...GEAGLEAG....LEAGEAGLEAGEAGLEAGEAGL.DDIKSPRLKAL
BTA112       ..............................KDPKEH...........DTESLNAKLDNL
BTA129       .....RFG......................REYTSKMVA............TGNNLDIKLNNL
```

```
                             α2                          α3               α4
fBTA121
                     190       200                           210       220
fBTA121      LDDFGVSRSGRKAISYIQGNLTS.............DVIYDRLNKLGADVVIEKIIKPT
BTA121       LDDFGVSRSGRKAISYIQGNLTS.............DVIYDRLNKLGADVVIEKIIKPT
BTA121-His   LDDFGVSRSGRKAISYIQGNLTS.............DVIYDRLNKLGADVVIEKIIKPT
BTA116       LDQFGVSGAGRKAVIYIRGGL.............LGDDVYDRLNKLGADVVIEKIIKPT
BTA123       LDQFGVSGPGRKAIGYIGGKFRDS............GDVHKRLNALGADVVIEKIIKPT
BTA126       LDNFGVSGAGKKTISYIQRTLPA.............NVVYDYLNQLGADVTIEKIIKPT
BTA127       LDQFGVSEAGRKAIGYIQGILRVS.......YKNGFDDKFYNCLNQLGADVVIEEIIKPT
BTA119       LDQFGVSGAGRKAISYIQGILSVS.......YKDGFDDKFYNCLNKLGADVAVEKIIKPT
BTA118       LDQFGVSGAGRKAISYIQGILRVS.......YKNGFDDKFYNSLNQLGADVAVEKIIKPT
BTA117       LDDFGVSGAGRKAIGYIQEILSFS.......YVDEFDDKFYNSLNKLGADVVVEKIIKPT
BTA122       LDQLGVSGAGRKAIGYIQGILSVS.......YKDGFDDKFYNCLNQLGADVVIEKIIKPT
BTA124       LDKLGVSGAGRKAIVYIRDNLTG..........DDVDDFFYNRLNKLRADIVIEKIIKPT
BTA120       LDQFGVSGAGRKAIGYIQEKLSVS.......YVDEFDDKFYNYLNKLGADVVIEKIIKPT
BTA115       LDDFNVSIEGKKAIGYIRKIFID........KNNDIEDKLYKHLNSLEADVVLAKIIKPT
BTA112       LNTFGISEAGKRSIVRIKDVVTDVGIGSVEGYRNYTDSELYALLNDLGAAK.IQEIIKAD
BTA129       LDKFGLLDKEREAIAYIRRTIITDSKIGNASYYKTYTDFEFYNLLESLGATR.IKEIITFY
```

```
               α5          η1                α6
fBTA121        230        240       250       260        270       280
fBTA121     VSLLKTKGEALKIIEDPTNEGVKTRLQNMCKRYDGLVKGIGY....DFFHGSIGTDRFAQ
BTA121      VSLLKTKGEALKIIEDPTNEGVKTRLQNMCKRYDGLVKGIGY....DFFHGSIGTDRFAQ
BTA121-His  VSLLKTKGEALKIIEDPTNEGVKTRLQNMCKRYDGLVKGIGY....DFFHGSIGTDRFAQ
BTA116      VSLLKARGEALRVIQDPTNEGVKVRLQDVFHRYDNLV.DIRG....KFIYVVGSEDNFAK
BTA123      VSLLKARGEALKVKEDPTNESIKTRLQDMLNRYDTLV.ESVWHD..FFHNRLIEEDMFVE
BTA126      VSLLRARGKALRVIEGTTDEGIKTGLQDMFEKYDALSFSYLWEV....FNRAIKENDFVE
BTA127      VSLLRARGEALRVIEGTTDEGVKTRLQDMLSSCDNVI.SFAWNP..DRDHLIFNEDKFAN
BTA119      VSLLIARGKALRVIQDPTNAGVKSRLQDMLSSCDHVI.SLTWYLISGFDYFIFEEDNFAK
BTA118      VSLLRARGEALRVIQDPTNAGVKSRLQDMLSSCDHVI.SLSWYLISGFDYFIFKEDVFTR
BTA117      VSLLRARGKALRVMQGTTDAGVKTRFQDMLSSCDHVV.SFTWYL..DFDHLSFREDIFAD
BTA122      VSLLRARGKALRVMQGTTDAGVKTRLQDMLSSCDLSV.SLAWYL..NSNPLIFHEDGFAD
BTA124      VILLRARGEALRVIEGTTDEDLKSKLRLVFHRYDSLV.....NIREKFNILLLERDIFEK
BTA120      VSLLIARGKALRVIQGITDADVKTRLQDMLSSCDYVV.SFTWYV..NFNPLIFKEDVFTR
BTA115      VSLLRARGEALRVIQDPTNESIKSRLQDVYDRYDALVKREFK...RYFVDRFGLIDSVVD
BTA112      LELVKTQKTALEAINNVHKPKERQNLQLRFNSKRNEY...PLH.....LKGLFNESDFNV
BTA129      LEVVNIQKSFERVIKNVKDATSRGKLQNELNERKNQY...QLH.....LKGLFDSDNFDD

            α7      η2   α8     α9      η3    η4        α10
fBTA121             290       300       310       320
fBTA121     A.....VVYYAPRFRKFKEIVKNPRVMDDIYGWLDADDRATINEIGKIVINA........
BTA121      A.....VVYYAPRFRKFKEIVKNPRVMDDIYGWLDADDRATINEIGKIVINA........
BTA121-His  A.....VVYYAPRFRKFKEIVKNPRVMDDIYGWLDADDRATINEIGKIVINA........
BTA116      A.....VTRYASKFSKFKEMVKNPRVM.DVYAWLDDADQATIDEIENIVISD........
BTA123      S.....VTYYVPKFSKFKEMVKHPRVI.DVYAWLDADDCVIIDEIEKIVVNA........
BTA126      F.....IARCDSRFRKLKEMVNSPRVK.DVYAWLDADDRVTIDDIGKIVISG........
BTA127      A.....VNRYTPKFSKFKEMVKNPRVK.DIYVWLDADEQATIDEIGNIVIST........
BTA119      A.....VTRYTPKFSKFKEMVKNPRVM.DIYVWLDADEQAIIDDMEKIVVNA........
BTA118      M.....ITRYTPKFSKFKEMVKNPRVM.DIYVWLDADEQAIIDDMEKIVVNA........
BTA117      L.....ITRYTSKFSKFKEMVKNPRVM.DIYVWLDADEQAIIDEIEKIVVNA........
BTA122      L.....ITRYTSKFSKFKEMVKNPRVK.DMYAWLDADEQAAIDEIEKIVVNA........
BTA124      T.....VTRYAPKFRKFKEMVTNPRLM.DMYAWLDADERATIDEIEKVVVNF........
BTA120      I.....ITRYTSKFRKFKEMVKNPRVM.DVYVWLDADEQAAIDEIEKIVVNA........
BTA115      T.....VTSCTSKFRKFKEMVKSPRVM.DVYGWLDVDEQATINEIEKIVVNA........
BTA112      VYSNVIGDNYVNEFTAIKEEIIQVIGDFDLYKGLSSREKSVVDYIQSVVTNANFCYANND
BTA129      IYNEIIGDNYFSELIKFKDEITKVEGGLDVYTWLSDDEQIVIDKIRGIVTDPLIG.KYKG

                                       α11
fBTA121     330       340       350       360            370       380
fBTA121     ..TYDKDKFNNVLNSVGVYYVVRMIDIYRGVKIEHDEA....LNAITTVPDGVVKQDLQA
BTA121      ..TYDKDKFNNVLNSVGVYYVVRMIDIYRGVKIEHDEA....LNAITTVPDGVVKQDLQA
BTA121-His  ..TYDKDKFNNVLNSVGVYYVVRMIDIYRGVKIEHDEA....LNAITTVPDGVVKQDLQA
BTA116      ..TYDKDKFNSMLNSLDDYRIVRIIDIYRDIKIKQEEA....LKAIEGVSDDAEKQNLKT
BTA123      ..TYDQDRFNNMLNSLGDANVIAIIKIYRDIKIEQGEA....LKAIDSISDDIVKQAYQD
BTA126      ..TYDKDRFNNMLNLLEDPLVIKIVVLCQSIKKRQEEA....LKAIEGVSDDAEKQKLQA
BTA127      ..TYDKDKFNNVLNSVSDYHVVKIISFCRLVKKMQEEA....LNAIEGVSDDAEKQKLQV
BTA119      ..TYDKEKFNDKLNSLSDYRVAEIVYNYRDIKNGREEA....LKAIDGVSDDAEKQNLKT
BTA118      ..TYDKEKFNDKLNSLSDYRVAEIVCYYRDFKNDQEEA....LKAIEGVSDDAEKQNLKT
BTA117      ..TYDKDKFNKKLNSLSDYHVATIVYNYRDVKIKREEA....LKAIDGVSDDAEKQNLKT
BTA122      ..TYDRDKFNKKLNSLSDYYVAAIVYSYRDIKNDQEEA....LKAVEGVSDDTEKQNLKA
BTA124      ..TYTKEKFDDILDNLSSGYIVKIIEIYEDIKIKQEEV....SKAIATVSDDFLRQDLQA
BTA120      ..TYDKEKFNDKLNSLSDYRVAKIVCYYRDFKNDQEEA....LKAIEGVSDDAEKQNLKT
BTA115      ..TYDKDKFNKKLNSIDDFNIFELIQACKKVLIEKKAA....DEAINGVSDDAEKQRLRN
BTA112      CKTYTLTEFYALLNSLGDAKIREMIKVNLDVFKVREEI....EADIAGLTEEKVKQALKT
BTA129      YRTYTDIEFNNLLNELGDNKVFAMILVYLEEQSNQDAAWLEALEAIDSVDDADFREQLRV

                  α12                              α13
fBTA121         390       400       410       420       430       440
fBTA121     RLNRFKGEYYSNIRGTFK.GFTDGLHFQIMTDGDKYRNYFIILKFDAQAARVAKARGATG
BTA121      RLNRFKGEYYSNIRGTFK.GFTDGLHFQIMTDGDKYRNYFIILKFDAQAARVAKARGATG
BTA121-His  RLNRFKGEYYSNIRGTFK.GFTDGLHFQIMTDGDKYRNYFIILKFDAQAARVAKARGATG
BTA116      RLERLQGEYNSHIRDAFN.KSEGELSFQLTNDSNKYRRGFDVIKSNAKAVKASGAAGGGS
BTA123      RFNTLQGEYDSHIRDAFN.KASGELYAQIIGNGDKYRNDFIGSRNNARAAKFDEEAAAAA
BTA126      RFNTLQGEYNSHIRSAFN.QSSVDLYFQITGNGDKYRNDFNAIEKAAKAAEAAQGAAAAV
BTA127      IFNRLKDEYNSHIRDTFNQ.SSDKFYAF.FIKIIKYSNGFSILKEDAKKAVLRAAAAAKA
BTA119      RFNTLQGEYYSSIRDAFN.KSADDLYAQLSYYPGKYSSGFPKIRDDAKRAAAAAKEAAA.
BTA118      RFNTLQGEYYSSIRDAFN.KSADDLYAQLNHYPDEYSSGFPKIRDDAKRAAAAAKEAEA.
BTA117      RFNTLQGEYYSSIRDAFNNKSADGLYAQLGRDLYEYSSGFPKIRDDARRAAKAKAAEA.
BTA122      RFNMLKDEYYSHIRDAFN.RSSDDLYFQLNHYSYEYSSGFFKIKEDAKKAAAAAEAAK..
BTA124      RLKRVKDEYNADIRDAFD.KPLVELVRQILNNRDVYLGRLNGIKATVAVGKSEEEA....
BTA120      RFNTLQGEYYSHIRDAFN.KSADDLYSQLNRYPDEYSSGFSEIKEDAKKAAAAAAAAERA
BTA115      NFNELKGMYYSHIRDAFA.LSIDELCYAIEHYHNNYYADFYGIRVET.............
BTA112      RLASYNNEYAFILRGAFN.ASGADNIYSRFMKI.NYINAYINLKKIRDP.RVLDVYTWL
BTA129      KIDDERDNYKLHFKELFN.EFLSELVYQKFMKNICYYDKFVLIKTAQHIIAFKKLYKKL
```

fBTA121

```
fBTA121      SG.S........................................................
BTA121       SG.S........................................................
BTA121-His   SG.S........................................................
BTA116       ............................................................
BTA123       KE.AKEAEEAAAA.........A..ERAKEAKEAEAAKAAEAAKRAKEAKEAEAAAAAKR
BTA126       KS.SGA......................................................
BTA127       SG.AAG.............................................GS.......
BTA119       ............................................................
BTA118       ............................................................
BTA117       ....K.......................................................
BTA122       ............................................................
BTA124       ............................................................
BTA120       KE.AKE.............................................AAVAAE...
BTA115       ............................................................
BTA112       SDEDKGVIDKIQGIVTDSNIGHAKGYKTYTDSEFNDLLNNLGASKTRSMIT.....AFLR
BTA129       SSVQKRGLDYIRIVVTNFNIGVL..HKTYNAIEFESLLGSLGATRVERIID.....FHLN
```

fBTA121

```
fBTA121      ............................................................
BTA121       ............................................................
BTA121-His   ............................................................
BTA116       ............................................................
BTA123       AKEAKEAEAAAAAKVEESTEAGEPREGSGTDEESGATGSGS...................
BTA126       ....................................AGGGS...................
BTA127       ........................................S...................
BTA119       ..................AKAAKAEEGSGTDEESGAADGGS...................
BTA118       ..................AKAAEAEEGSGTDEESGAADGGS...................
BTA117       .............EAAVAAEAGEPREGSGTDEESGAADGGS...................
BTA122       .......................AEEGTKTDEESGTADGGS...................
BTA124       .....DKAAAAAVKAEGSTEAGEPGEGSGTVEESGATGGGS...................
BTA120       .....AEESTEAGEAEESTEAGEPREGSGTDEESGAADGGS...................
BTA115       ............................................................
BTA112       ANVLREM....ALEAINTVPAGTAKQNFQ..NRFNALSNGYALRLKGLFNSLNHDMIYDS
BTA129       VLKAKKD....AQSAISSLPESMTRHNLQ..RLFDAHSNGYELHLKELFHINFPYQVYYS
```

fBTA121

```
fBTA121      ................................
BTA121       ................................
BTA121-His   ................................
BTA116       ................................
BTA123       ................................
BTA126       ................................
BTA127       ................................
BTA119       ................................
BTA118       ................................
BTA117       ................................
BTA122       ................................
BTA124       ................................
BTA120       ................................
BTA115       ................................
BTA112       VVNDEYSEKFLRIKDDIRHLSSNTADSTASSN
BTA129       VARSSYVDLYFEIINYVGKIQ...........
```
